# Assessment of Nutrient Intakes: Introduction to the Special Issue

**DOI:** 10.3390/nu8040184

**Published:** 2016-03-25

**Authors:** Sharon I. Kirkpatrick, Clare E. Collins

**Affiliations:** 1 School of Public Health and Health Systems, University of Waterloo, Waterloo, ON N2L 3G1, Canada; 2 Priority Research Centre for Physical Activity and Nutrition, School of Health Sciences, Faculty of Health and Medicine, University of Newcastle, Newcastle, NSW 2308, Australia

Accurately measuring consumption of food, drinks and supplements is fundamental to nutrition and health research, including surveillance, epidemiology and intervention studies. However, assessing food intake is an area that is fraught with challenges [1]. Diet is inherently complex given that it is a chronic and multifaceted phenomenon that changes over time and varies in relation to age, life stage and many other factors. The challenges associated with assessing diet and nutrient intakes have led to the productive area of research that is the focus of this issue of *Nutrients*.

Usual diet, or long-term average diet, is the phenomenon typically of interest in nutrition and health research. However, objective measures of usual diet are few and can be of limited use, while carrying high costs and substantial researcher and respondent burden [2,3]. As a result, researchers typically rely on self-report measures, such as 24-h recalls, food records, food frequency questionnaires, and brief instruments. The extent of error in dietary data collected using self-report instruments and tools has recently been debated [4,5,6,7,8,9], with some critics suggesting that such data be abandoned. However, this error and its implications for interpreting the findings from nutrition research have long been recognized. Indeed, nutritionists, statisticians and epidemiologists in various parts of the world have made important advances over several years to better understand measurement error within self-reported dietary data and to identify ways to address it. These advances have included the use of recovery biomarkers, which provide an objective measure of true intake for energy and a few nutrients, to ascertain the types and degree of error affecting data collected using different types of instruments, e.g., [10,11]. The findings of biomarker-based studies have informed recommendations for measuring diet in different types of studies, e.g., [1] and the development of statistical methods that allow for adjustment for error, e.g., [12,13,14,15,16]. There has also been work to combine concentration biomarker and self-report data to mitigate measurement error, e.g., [14,17]. Further, the evolving understanding of limitations of existing tools combined with technological advances have enabled a new generation of dietary assessment instruments and strategies, including web-based tools, mobile apps, and image-based assessment, which are aimed at mitigating some of the challenges of traditional methods and modes of data collection [3].

This Special Issue of *Nutrients* provides examples of the diversity of research that is underway internationally to advance the robust collection and use of dietary data. The issue encompasses 27 articles (18–44), with contributions from Australia, Brazil, Canada, Guam, Japan, Russia, South Korea, the United Kingdom, and the United States, as well as several countries in the European Union. The range of articles included demonstrates efforts being made to improve the quality of dietary data collected from populations across the lifecycle and across contexts, with applications related to characterizing dietary intakes, as well as associations between diet and health. Articles in this issue describe the development and/or evaluation of new tools [18,19,20,21,22,23,24,25,26,27], some of which take advantage of technology to address limitations in existing tools. Among these are mobile phone instruments [21,22,25], including a novel tool that incorporates not only health but also sustainability considerations related to diet [23], as well as a web-based 24-h recall developed in the UK [24]. The use of biomarkers to assess intake and to evaluate self-report tools is also highlighted. For example, Tasevska examines the evidence to support the use of urinary sugars as a biomarker for total sugar intake [28], Lai *et al.* examine the use of biomarkers to assess the validity of a food frequency questionnaire [20], and Zheng *et al.* explore the potential of metabolomics as markers of intake [29].

The issue also highlights efforts to better understand the optimal use of existing tools. For example, Kerr *et al*. examine whether accuracy of recall among adolescents improves with a second administration of a 24-h dietary recall [30]. Of interest in terms of understanding the comparability of data generated by surveillance systems in different countries, De Keyzer *et al.* [31] provide a narrative review of methods used to assess diet in national food consumption surveys across continents. Such reviews and consideration of their findings are increasingly important as it is recognized that inconsistency in assessment methods complicates the interpretation of the larger evidence base, as well as posing a barrier to efforts to conduct pooled and cross-country analyses. Statistical methods for estimating usual intake of dietary components are also addressed, with a comparison of methods undertaken by Laureano *et al.* [32]

Finally, applied papers demonstrate the ways in which existing methods and resulting data contribute to our understanding of diet among populations [33,34,35,36,37,38,39,40], as well to the evaluation of interventions [41]. Papers within this issue also address dietary patterns and diet quality indices [42,43,44], growing areas within nutrition research as the importance of embracing the complexity of what we eat and drink is increasingly recognized.

This collection of papers demonstrates that dietary assessment research is alive and well. The advances in the use of technologic innovation and biomarkers to enhance measures of diet have the potential to contribute to the collection of higher quality data in future research. This issue of *Nutrients* also provides an opportunity to consider gaps in the evidence base on the assessment of diet and future research directions that could potentially benefit from a more collaborative approach. Working together across institutions and countries can provide researchers with the opportunity to learn from one another, as well as to leverage scarce resources to advance the field.
